# Maternal Voice and Tactile Stimulation Modulate Oxytocin in Mothers of Hospitalized Preterm Infants: A Randomized Crossover Trial

**DOI:** 10.3390/children10091469

**Published:** 2023-08-28

**Authors:** Jessica Hirschel, Audrey Carlhan-Ledermann, Céline Ferraz, Laure-Anne Brand, Manuela Filippa, Edouard Gentaz, Fleur Lejeune, Olivier Baud

**Affiliations:** 1 Division of Neonatology and Pediatric Intensive Care, Children’s University Hospital of Geneva, University of Geneva, 1205 Geneva, Switzerland; jessica.hirschel@hcuge.ch (J.H.); audrey.ledermann@hcuge.ch (A.C.-L.); celine.ferraz@hcuge.ch (C.F.); laure-anne.brand@hcuge.ch (L.-A.B.); 2 Division of Development and Growth, Department of Pediatrics, University Hospital of Geneva, 1205 Geneva, Switzerland; manuela.filippa@unige.ch; 3 Department of Psychology and Educational Sciences, University of Geneva, 1211 Geneva, Switzerland; 4 Sensorimotor, Affective and Social Development Unit, Faculty of Psychology, University of Geneva, 1211 Geneva, Switzerland; edouard.gentaz@unige.ch (E.G.); fleur.lejeune@unige.ch (F.L.); 5 Inserm U1141, University of Paris, Paris 75019, France

**Keywords:** oxytocin, preterm infants, infant-directed speech, maternal voice

## Abstract

Prematurity is a major risk factor for perinatal stress and neonatal complications leading to systemic inflammation and abnormal mother–infant interactions. Oxytocin (OT) is a neuropeptide regulating the inflammatory response and promoting mother–infant bonding. The release of this hormone might be influenced by either vocal or tactile stimulation. The main objective of the current randomized, crossover, clinical trial was to assess the salivary OT/cortisol balance in mothers following the exposure of their baby born preterm to two types of sensorial interventions: maternal voice without or with contingent tactile stimulation provided by the mother to her infant. Among the 26 mothers enrolled, maternal voice intervention alone had no effect on OT and cortisol levels in the mothers, but when associated with tactile stimulation, it induced a significant increase in maternal saliva oxytocin (38.26 ± 30.26 pg/mL before vs 53.91 ± 48.84 pg/mL after, *p* = 0.02), particularly in the mothers who delivered a female neonate. Maternal voice intervention induced a significant reduction in cortisol and an increase in OT levels in mothers when the maternal voice with a tactile stimulation intervention was performed first. In conclusion, exposure to the maternal voice with a contingent tactile stimulation was associated with subtle changes in the maternal hormonal balance between OT and cortisol. These findings need to be confirmed in a larger sample size and may ultimately guide caregivers in providing the best intervention to reduce parental stress following preterm delivery.

## 1. Introduction

Prematurity is a major risk factor of neurocognitive impairment and neurobehavioral disorders during infancy and in adults [1]. While the survival of preterm infants is increasing, ~10% of the 1.6 million infants born very preterm each year develop cerebral palsy, and ~35% have persistent cognitive and neuropsychiatric deficits [2]. There is strong evidence that an adverse environment during pregnancy and in the perinatal period can influence hormonal responses to neonatal stress, with long-lasting neurobehavioral consequences in infancy and adulthood [3,4,5].

The beneficial effects of exposure to the maternal voice on the physiological and behavioral constants of hospitalized stressed preterm infants were demonstrated [6,7], but their hormonal effect in their mothers remains still under investigated [8]. The neuropeptide oxytocin (OT) plays an important role in the perinatal period, and its importance for lactation and social bonding in early life are well recognized [9], especially in conditions of pain and separation [10]. Recently, OT was identified as a key hormone to alleviate anxiety in parents and in infants subjected to pain [8], but data regarding OT response to maternal sensory interventions under basal conditions are still lacking. In addition to its peripheral action when released into the systemic circulation, OT, synthesized by the hypothalamus, acts as a neurotransmitter when it is involved at the brain level. In the neonatal intensive care unit (NICU), Weber et al. reported the developmental trajectory of OT in premature babies and showed a spontaneous decrease in one-week increments [11]. Its synthesis was notably studied to identify the biological effect of skin-to-skin carrying, where it increases in both parents and children [12]. Other studies assessed its relationship with parental attachment and showed conflicting results [13,14,15]. In addition to its effect on maternal behavior, other roles of OT were demonstrated, including its anti-inflammatory properties both in the developing and mature brain [16] and a modulatory effect on the hypothalamo-pituitary and adrenal axis. OT was reported to induce a reduction in corticotrophin-releasing hormone mRNA levels in the paraventricular nucleus in response to stress [17,18] and a reduction in adrenocorticotropic hormone and corticosterone plasma levels both in the basal condition and in response to stress [19,20]. These elements would support a neuroprotective effect of OT.

Early multisensory interventions in the NICUs improved several aspects in infants’ development and reduced maternal stress, but not all findings were consistent [21]. Premature infants show clear differences in brain responses to multisensory stimuli when compared to terms, and these differences are predictive of long-term difficulties in sensory functions and internalization [22].

In the neonatal period, the optimal conditions of exposure to human voice, live or recorded, of hospitalized preterm infants are not yet clearly established. Previous research showed that the presence of a recorded female voice interfered with tactile sensory learning in preterm human infants [23]. The authors hypothesized that the perception of the contingency of two events is essential to learning and memory from early infancy, since it gives a coherent sense of the multisensory environment [24].

The primary objective of this research was to investigate if a live maternal voice was associated with changes in OT and cortisol saliva concentrations in mothers of preterm infants and if a concomitant tactile stimulation would modulate this hormonal response.

## 2. Methods

### 2.1. Patients and Their Characteristics

We conducted a randomized, crossover, monocentric, unmasked clinical trial (NCT04665440) that enrolled 28 mothers who delivered neonates between 32 and 35 weeks’ gestation. Infants were aged from 5 to 12 days at the interventions and were free of morbidities or respiratory support. For multiple pregnancies, the mother was investigated only once, and interventions were applied to the first newborn. The study was approved by the Geneva Research Ethics Committee (BASEC No. 2020-00101) and was audited by the clinical research center, University Hospitals Geneva. A written consent was collected from the parents of each infant. Data on maternal characteristics and pregnancy events were extracted from the clinical database of the perinatal center. Maternal characteristics included demographic data, the main cause of preterm delivery, antenatal steroids, and delivery route. Neonatal characteristics of the infant included the sex, gestational age at birth, birth weight, intra-uterine growth restriction (defined as birth weight < 10th percentile), APGAR score at 5 min, and potential complications of preterm birth that occurred before the interventions. Early onset sepsis (EOS) was defined as the association of biological signs and clinical deterioration, with or without bacteriological documentation before 48 h of life. Transient tachypnea of the newborn was defined as supplemental oxygen or respiratory support for less than 72 h after birth.

### 2.2. Study Design and Interventions

**Figure 1:**
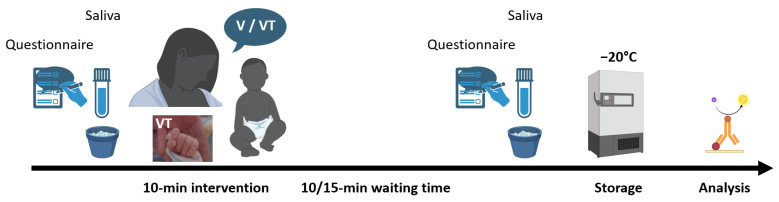
Timeline of the study. After consent was obtained, the recruited mothers were invited to expose their baby to two interventions whose order was randomly allocated and performed once in the afternoon during two consecutive days: the maternal voice intervention (V), and the maternal voice with a third-party tactile stimulation intervention (VT). Maternal saliva was collected just before and 10–15 min after each intervention by passive drool and immediately frozen at −20 °C. Saliva oxytocin was measured by quantitative electro-chemoluminescence and saliva cortisol by enzyme immune assay. Mothers were asked to fill in the PSS-NICU questionnaire just before the first intervention and just after the second intervention.

In this crossover trial, the infants of the recruited mothers were exposed to two interventions whose order was randomly allocated and performed once in the afternoon during two consecutive days (Figure 1): maternal voice exposure and maternal voice exposure associated with a third-party tactile stimulation. Each intervention lasted 10 min and no other medical exam was planned on the day of study interventions. Each infant was installed in his incubator in a state of quiet wakefulness. The mother sat next to the incubator so she could see and spoke to her infant, but she was out of her infant’s field of vision to minimize visual stimulation and was instructed to avoid touching her baby.

For the maternal voice intervention (V), the mother was free to choose the contents and the language of her speech (minimum 10 dBA above the background noise).

For the intervention combining the maternal voice and a third-party tactile stimulation (VT), the experimenter was standing on the other side of the incubator facing the mother, out of the field of the infant’s vision. While the mother spoke to her infant, the experimenter placed a wooden object (prism or cylinder) in the infant’s hand (for details about these stimuli, see [25]). When the infant released the object, the experimenter presented the second object. The prism and the cylinder were presented alternatively during the whole intervention to avoid habituation. A third-party was chosen to ensure that the tactile stimulation was standardized and induced distraction to the baby only without interference of the content of the mother’s speech. In addition, we also would make sure that the mother was really focused on the newborn response to her speech.

### 2.3. Sample Size Calculation

In a previous study [8], a sample size of 20 mothers who were asked to speak to their preterm infants in the incubators for 5 min before and after the heel-prick procedure was found to be associated with a significant effect on OT saliva concentration.

Here, under baseline conditions (without any painful procedure), we primarily hypothesized that the effect of a maternal voice on the OT saliva concentration in mothers will be similar. Considering the hypothesis that tactile stimulation of the baby may attenuate this effect by 50%, a total of 80 participants should be recruited. Taking into account the missing data and dropped out, we planned to include 84 children in total, i.e., 42 children per group.

### 2.4. Primary and Secondary Outcomes

The primary objective of this research was planned to investigate if a live maternal voice was associated with changes in saliva concentrations of cortisol and OT in preterm infants and their mothers. The secondary objective was to assess if a concomitant tactile stimulation would modulate this hormonal response in the mother–neonate dyads.

The primary outcomes were planned to be biological measurements of saliva OT and cortisol for both the mother and the newborn. Due to technical issues in collecting an appropriate volume of the infant’s saliva, maternal samples only were processed. Maternal saliva (500 µL) was collected at a distance (>2 h) of foods with high sugar, acidity, or caffeine before and 10–15 min after each intervention. Following passive drool into a validated collection device, samples were placed on wet ice immediately, and frozen and stored at −20 °C within 30 min. Saliva OT was measured in triplicate by quantitative electrochemoluminescence with a sensitivity of 4 pg/mL (assay range of 8–1000 pg/mL) by Salimetrics (Carlsbad, CA, USA). Saliva cortisol was measured in duplicate by enzyme immune assay with a sensitivity of less than 0.007 µg/dL (assay range of 0.012–3.000 µg/dL).

The mothers were invited to fill in a questionnaire just before the first intervention and just after the second intervention. The questionnaire was the Parental Stressor Scale Neonatal Intensive Care Unit (PSS-NICU; [26]). It includes 34 items divided into three subscales (parental role restriction, sights and sounds in the NICU, and infant appearance and behavior). The degree of stress is determined in a 5-point scale (1 = least stressful to 5 = most stressful). The total score is computed by calculating the mean of the 34 items, as well as the mean score for each subcale. A higher total score indicates greater overall stress.

### 2.5. Statistical Analysis

Qualitative variables were described as frequencies (percentages) and quantitative variables as mean (standard deviation) and median (first–third quartiles [IQR]). Group comparisons were computed using either Fisher’s exact test for qualitative variables and Mann–Whitney tests for quantitative variables. Non-parametric Wilcoxon–Mann–Whitney paired tests were used for comparing quantitative biological variables between two groups. When comparing more than two groups, either a Kruskal–Wallis or a Friedman test of repeated measures of one-way ANOVA was used, followed by a Dunn’s multiple comparisons test when appropriate. Because the first intervention may affect the result of the second intervention, the carryover effect was addressed using a mixed-effects model for both OT and cortisol changes before and after intervention, according to the sequence used (V then VT or VT then V). The effect of the sequence randomly assigned to the subjects and sequence x intervention interaction were tested. For all analyses, *p*-value < 0.05 was considered significant. Statistical tests were run on GraphPad Prism version 9.00 (GraphPad Software, San Diego, CA, USA).

## 3. Results

**Figure 2:**
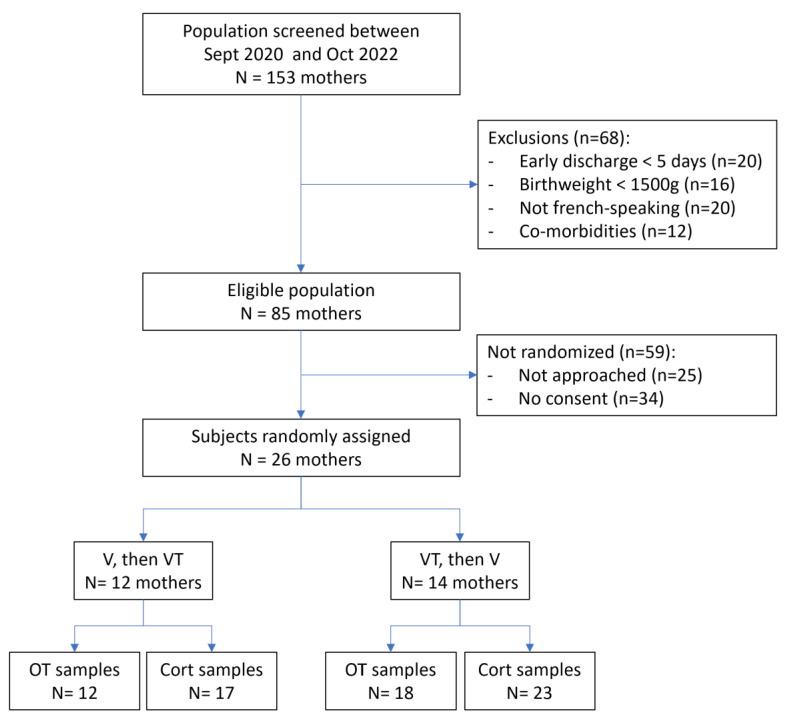
Flow chart of the study.

The flow chart of the study is described in the Figure 2. Among 153 mothers initially screened at Geneva Children’s Hospital, 28 were enrolled in the study. One of them withdrew consent in each arm.

V then VT means that maternal voice alone was performed the first day and maternal voice with a third-party tactile stimulation the day after.

VT then V means maternal voice with a third-party tactile stimulation was performed the first day and maternal voice alone the day after.

OT means oxytocin.

Cort means cortisol.

Twelve mothers were assigned to the group for which the vocal intervention alone should be conducted first, and fourteen assigned to the group for which intervention combining vocal exposure and tactile stimulation should be conducted first. Characteristics of the studied population are described in Table 1. Overall, infants delivered from these recruited mothers had a median gestational age at birth of 34 weeks and the most frequent co-morbidities associated with prematurity were jaundice treated by phototherapy and transient tachypnea of the newborn. No neonatal brain lesion was reported.

**Figure 3:**
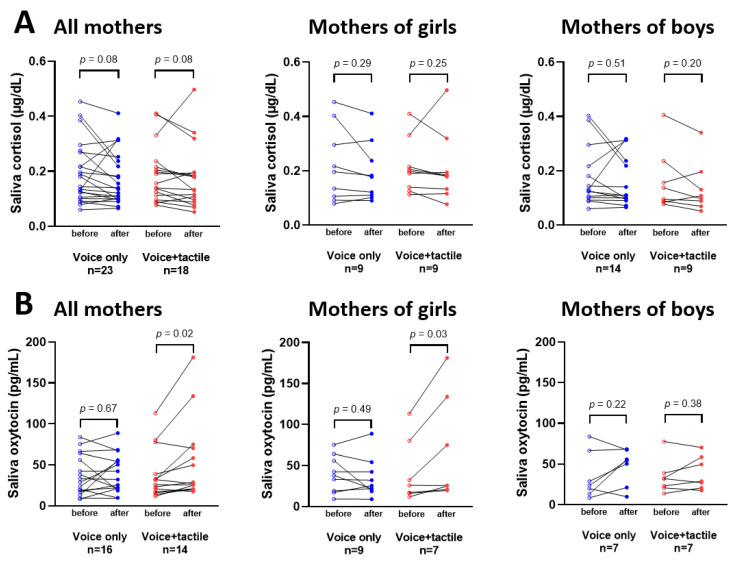
Cortisol (**A**) and oxytocin (**B**) salivary concentrations in mothers before and after sensory stimulation, according to the intervention group and the sex of their neonates. Non-parametric Wilcoxon–Mann–Whitney paired tests were used for comparing quantitative biological variables between two groups.

We first assessed the ability of each intervention to induce changes in saliva concentrations of cortisol and OT in the mothers. Figure 3 shows for each subject the effect of each intervention, maternal voice only (V) and maternal voice with third-party tactile stimulation (VT), on saliva concentrations of cortisol and OT in the mothers. Maternal voice intervention was associated with no statistically significant change in both hormones when measured in mother’s saliva before and 10–15 min after the intervention. In contrast, maternal voice with third-party tactile stimulation induced a significant increase in maternal saliva oxytocin (mean ± standard deviation: 38.26 ± 30.26 pg/mL before vs. 53.91 ± 48.84 pg/mL after; mean difference [95% confidence intervals]: −2.5 [−17.8 to 12.9] before vs. 15.6 [2.2 to 29.1] after; *p* = 0.02), particularly in the mothers who delivered a female neonate. Cortisol levels were found to be similar before and after the combined intervention, independently of the sex of the newborn.

Next, we studied a potential carryover effect induced by the crossover design by assessing hormonal changes according to the order of each intervention. Table 2 describes the mean (SD) and median (IQR) values of OT and cortisol saliva concentrations before and after each intervention (V or VT) according to their order in the protocol.

**Figure 4:**
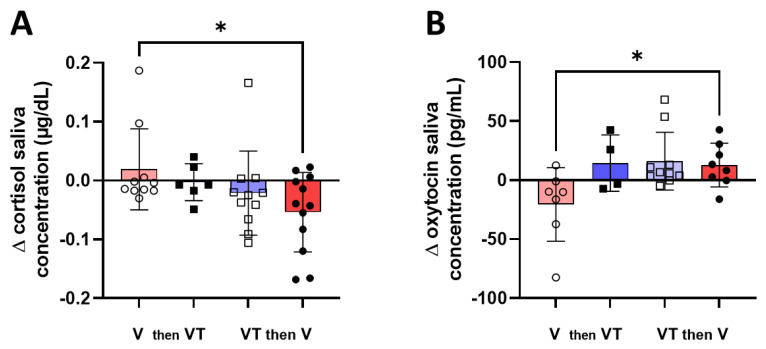
Changes in cortisol (**A**) and oxytocin (**B**) salivary concentrations according to the order of each intervention (maternal voice first or maternal voice + tactile stimulation first). A Kruskal–Wallis test followed by a Dunn’s multiple comparisons test were used. * means *p* < 0.05.

Whether the order of the two interventions may induce a different modulation of cortisol and OT concentrations as measured in the mother’s saliva is depicted in Figure 4. We found that the maternal voice with third-party tactile stimulation induced a similar effect on hormone concentrations whether this intervention was performed on Day 1 or Day 2. In contrast, both a statistically significant reduction in cortisol level (mean ± standard deviation: 0.019 ± 0,069 vs. −0.054 ± 0,067 µg/dL; mean difference [95% confidence intervals]: 0.019 [−0.030 to 0.068] first vs. −0.054 [−0.096 to −0.011] second, *p* = 0.048) and an increase in OT level (mean ± standard deviation: −20.61 ± 31.19 vs. 12.89 ± 18.55 pg/mL; mean difference [95% confidence intervals]: −20.6 [−49.5 to 8.2] first vs. 12.9 [−2.6 to 28.4] second, *p* = 0.01) was observed when maternal voice without tactile stimulation was performed on Day 2 (Figure 4).

In addition, the period effect (V then VT vs. VT then V) and the period x intervention effect interaction were assessed using mixed-effects models for both OT and cortisol. These analyses failed to demonstrate any significant effect of either period or interaction for OT (*p* = 0.10 and *p* = 0.14, respectively) and for cortisol (*p* = 0.94 and *p* = 0.43, respectively).

**Figure 5:**
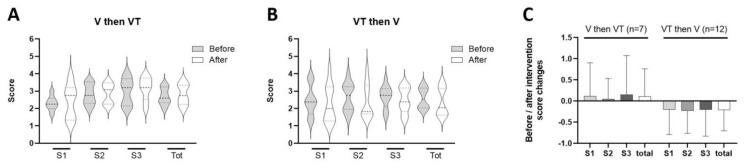
PSS-NICU questionnaire score and subscores just before and after the two cycles of interventions according to the order of each intervention (**A**,**B**). Mean differences calculated between scores assessed after the interventions compared to those assessed before were depicted in (**C**). Error marks show standard deviations.

Finally, mothers were invited to fill in the PSS-NICU questionnaire to assess their degree of stress just before and after the two cycles of interventions (V and VT, in a random order). PSS-NICU provides an overall stress score and subscores focused on sights and sounds in the NICU (S1), infant appearance and behavior (S2), and parent role restriction (S3). No significant difference was observed among these scores and subscores, before and after the interventions (Figure 5A,B). However, a trend towards lower scores was observed in each subscore and total score of the PSS-NICU for mothers who performed the maternal voice intervention the second day; this finding is consistent with hormonal changes (lower cortisol and higher OT levels) observed in the same condition (Figure 4).

## 4. Discussion

This study reveals subtle changes in hormonal balance in mothers talking to their preterm baby in the stressful context of the NICU, with a notable increase in OT concentration in saliva when the maternal voice was associated with contingent tactile stimulation. Randomization of this trial appears to be quite balanced, but postnatal age at discharge significantly differs. This difference may be a consequence of the slightly lower gestational age at birth in the group with late hospital discharge and is certainly not a direct result of the interventions.

Despite an abundant literature on the role of socio-emotional environment and parental interactions in the neurodevelopmental outcomes of infants born preterm [27], only a few studies addressed the developmental trajectories of OT in the neonatal period and its balance with cortisol in dyadic interactive contexts [11,15]. In addition to the effect of skin-to-skin contact on OT salivary levels in neonates [12], and the effects of the maternal speech and singing during painful procedures [8], no other study addressed the ability of an early intervention to change OT levels in mothers.

Our findings are of interest to identify the optimal conditions of exposure to the human voice for hospitalized preterm infants. Indeed, exposure to a recorded female voice, other than the maternal voice, in preterm infants was shown to impair their handling of tactile abilities [25], suggesting a detrimental effect of the recorded voice in a specific context of sensory learning. In contrast, the present study reveals that the intervention including live maternal voice and a contingent tactile stimulation induced a significant increase in maternal saliva OT, but not the maternal voice alone. This result suggests that it could be more comfortable for the mothers not to be the center of attention because during this intervention, the experimenter was focused on the tactile stimulation. This context would allow the mother to feel more relaxed while talking to her infant. This interpretation is in line with our second result regarding the order of interventions. Indeed, when the maternal voice with tactile stimulation intervention is carried out on the first day, the beneficial effects of the maternal voice alone are then observed on the second day with an increase in OT and a decrease in cortisol. It seems important to offer mothers a secure multisensory environment so that they can interact positively and without stress with their infant. Our data also suggest that a sort of priming effect initiated by the tactile stimulation associated with the maternal voice the first day may lead to a more robust, coordinated cognitive and physiological response when another vocal stimulation occurs the second day. This potential priming was reported to change perceptual processing, consistent with predictive coding models of repetition suppression, leading ultimately to more efficient and rapid behavioral responses [28].

Moreover, the increase in maternal OT for the intervention including maternal voice and a contingent tactile stimulation was observed only for mothers of girls. This finding is in line with a recent paper showing that parvocellular OT neurons are primarily activated by somatosensory stimuli, transmitted to the larger population of magnocellular neurons, and consequently show coordinated increases in their activity during social interactions between virgin female rats [29]. This sex difference could also be explained related to specificities that preterm girls show in the neonatal period when compared to boys. Indeed, it was demonstrated that very preterm boys have greater alterations in resting neurophysiological network communication than girls [30]. However, to our knowledge, nothing was reported so far regarding the sex-dependent hormonal balance of the mothers of preterm infants.

Biological measurements of OT and cortisol were the primary outcomes of the present study because OT was shown to balance cortisol release in response to stress [18,31]. In addition, OT was reported to confer an important role in protecting the brain exposed to the pro-inflammatory effects of cortisol. In addition to its role in labor and lactation, recent preclinical investigations also evidenced a pivotal role of OT in regulating the central inflammatory response [16,32,33,34] and in modulating several behaviors, including social recognition, emotional disorders, and parenting [35]. Furthermore, OT is an important hormone for the regulation of maternal behavior and maternal care both in animal models [36,37] and in humans [38,39,40]. Early life stress disturbs attachment behavior [41], which in turn affects the endogenous OT system itself [42], leading to reduced cognitive performances and adult psychopathy [43,44]. Altogether, these data strongly support a role for OT as a modulator of central nervous system functions.

A recent study reported that salivary OT levels significantly increased from late pregnancy to 1 day postpartum and then decreased until 5 days postpartum, a decrease associated with maternity blues, postpartum fatigue [45], and that could be exacerbated by perinatal stress of preterm delivery. OT release can be reduced by stress, fear, and pain [46] mitigating activation of brain reward networks that promote maternal–newborn bonding and caretaking [47,48]. To restore these physiological processes, increasing evidence supports the beneficial effect of the direct intervention of parents towards their preterm babies [49]. However, the best interventions and their combination to reduce both parental stress and promote neonatal brain development remain to be determined. Recent animal studies showed the significant effect of social vocalizations on OT regulation and social behaviors [50,51], and that auditory exposure to music or voice also induces increased OT and decreased cortisol concentrations in human adults [52].

This study has several limitations. First, the sample size initially planned was not reached due to recruitment issues. Indeed, besides the COVID crisis that occurred during the recruitment phase, we did not anticipate that a substantial proportion of eligible subjects would not be French-speaking and that many infants would be transferred to another hospital early after birth. In addition, there was a relatively large proportion (40%) of parents who did not consent. The early termination of the trial reduced the ability to achieve adequate power for the study, which limits our ability to assess the effect of maternal voice on OT concentration and obviously precludes any definitive conclusion regarding the effect of a concomitant tactile stimulation. Second, only maternal samples met requirements for OT measurements because saliva volumes collected in neonates were too small. Dyad analyses of saliva hormonal changes were therefore not feasible. Third, some issues with OT measurement in saliva may relate to the reliability and accuracy of the method used. Indeed, the concentration of OT in saliva is relatively low, leading to potential inconsistencies in results, and other factors, such as the timing of sample collection, sample handling, and storage conditions, can significantly impact the measured OT levels. Some authors claimed that salivary OT has only little or no correlation with cerebrospinal fluid, as well as little relevance in terms of physiological function [53,54]. Conversely, another report showed that OT levels in saliva correlate better than plasma levels with concentrations in the cerebrospinal fluid of patients in neurocritical care [55]. Quantification of the OT level in the saliva is also considered to be a non-invasive measurement method in vulnerable patients [56]. Two previous studies showed the correlation between salivary and plasma OT levels in postpartum women [40,57]. Furthermore, alternative methods for measuring OT, such as blood sampling, while considered more reliable, require invasive procedures not appropriate in a study investigating how to reduce stress in a NICU. Measuring OT in saliva using chemoluminescence-based assays is a popular approach used in the present study. However, the need for further validation studies and the development of standardized protocols to ensure the reliability of these measurements was recently documented.

In conclusion, this study provides preliminary evidence that live maternal voice intervention to their preterm infants may influence hormonal balance in mothers towards higher OT/cortisol ratio under certain experimental conditions. These findings need to be confirmed in a larger sample size and correlated with the hormonal effect of these interventions on hormonal regulation in the neonate and ultimately on their neurodevelopmental outcomes.

## Figures and Tables

**Table 1:** Baseline characteristics of mother–neonate dyads.

Variable	V, Then VT (n = 12)	VT, Then V (n = 14)	*p*-Value *
Mothers			
Maternal age at delivery, years			0.35
median (IQR)	32 (30–38)	35 (33–37)	
mean (SD)	33 (8.5)	35 (4.5)	
Ethnicity			0.65
Caucasian	10 (83.3%)	10 (71.4%)	
Hispanic	1 (8.3%)	1 (7.1%)	
Asian	0	1 (7.1%)	
African	0	1 (7.1%)	
Not reported	1 (8.3%)	1 (7.1%)	
Educational level			0.31 **
Tertiary	6 (50.0%)	4 (28.6%)	
Upper secondary	1 (8.3%)	4 (28.6%)	
Lower secondary	5 (41.7%)	2 (14.3%)	
Primary	0	2 (14.3%)	
Not reported	0	1 (7.1%)	
Family structure			0.43
Married couple	5 (41.7%)	9 (64.3%)	
Not married couple	6 (50.0%)	3 (21.4%)	
Single	1 (8.3%)	1 (7.1%)	
unknown	0	1 (7.1)	
Singleton pregnancy	7 (58.3%)	9 (64.3%)	>0.99
Preterm rupture of membranes > 24 h	1 (8.3%)	1 (7.1%)	>0.99
Choriomamnionitis	0	0	-
IUGR < 10 perc.	1 (8.3%)	2 (14.3%)	>0.99
Preeclampsia	3 (25.0%)	2 (14.3%)	0.63
Gestational diabetes	1 (8.3%)	0	0.46
Vaginal delivery	6 (50.0%)	5 (35.7%)	0.69
**Neonates**			
Male sex	7 (58.3%)	7 (50%)	0.71
Gestational age at birth, weeks			0.13
median (IQR)	34.2 (34.0–34.3)	33.2 (33.0–34.2)	
mean (SD)	34.1 (0.4)	33.3 (1.4)	
Birthweight, g			0.59
median (IQR)	2183 (1754–2370)	1900 (1850–2163)	
mean (SD)	2080 (625)	1974 (313)	
Head circumference, cm			0.33
median (IQR)	30.0 (29.5–32.0)	31.5 (30.3–32.8)	
mean (SD)	30.6 (2.5)	31.3 (2.5)	
Apgar score at 5 min			0.89
median (IQR)	9.5 (7.8–10.0)	9.0 (8.0–10.0)	
mean (SD)	8.8 (2.3)	8.8 (2.0)	
Early onset sepsis	0	0	-
Hypoglycemia	2 (16.7%)	1 (7.1%)	0.58
Transient tachypnea of the newborn	8 (66.7%)	6 (42.9%)	0.27
Exogenous surfactant	0	0	-
Phototherapy	5 (41.7%)	5 (35.7%)	>0.99
Exclusive mother milk	3 (25.0%)	1 (7.1%)	0.31
Age at feeding autonomy, days			0.36
median (IQR)	13.0 (4.5–19.5)	19.0 (11.0–25.0)	
mean (SD)	13.6 (15)	18.0 (14)	
Abnormal cranial ultrasound	0	0	-
Age at hospital discharge, days			0.03
median (IQR)	21.0 (14.5–26.0)	31.0 (26.0–36.0)	
mean (SD)	21.5 (11.5)	41.1 (10.0)	

IQR means interquartile range; SD means standard deviation; IUGR means intra-uterine growth restriction; * Fisher’s exact test for qualitative variables and Mann–Whitney tests for quantitative variables; and ** chi-square test for trend.

**Table 2:** Cortisol and oxytocin saliva concentrations according to each intervention and its order.

Intervention Order	Cortisol (µg/dL)		Oxytocin (pg/mL)	
	Before	After	Before	After
**V first**	n = 11	n = 11	n = 8	n = 8
Median (IQR)	0.132 (0.107–0.217)	0.111 (0.097–0.253)	60.0 (23.3–79.4)	26.8 (12.2–64.1)
Mean (SD)	0.197 (0.183)	0.158 (0.091)	53.0 (29.7)	35.2 (24.8)
**VT second**	n = 6	n = 6	n = 4	n = 4
Median (IQR)	0.133 (0.094–0.168)	0.121 (0.086–0.186)	32.6 (23.7–66.4)	64.5 (27.8–73.8)
Mean (SD)	0.134 (0.041)	0.131 (0.049)	40.9 (25.1)	55.4 (26.1)
**VT first**	n = 11	n = 11	n = 10	n = 10
Median (IQR)	0.206 (0.113–0.332)	0.179 (0.097–0.320)	24.5 (15.6–49.2)	26.5 (20.9–70.8)
Mean (SD)	0.220 (0.119)	0.199 (0.136)	37.2 (33.3)	53.3 (56.7)
**V second**	n = 12	n = 12	n = 8	n = 8
Median (IQR)	0.207 (0.122–0.364)	0.167 (0.101–0.233)	26.3 (17.8–41.4)	46.4 (22.9–55.5)
Mean (SD)	0.237 (0.129)	0.183 (0.100)	32.3 (20.1)	45.2 (22.7)

IQR means interquartile range; SD means standard deviation.

## Data Availability

The data presented in this study are available on request from the corresponding author.
